# The use of entertainment and communication technologies before sleep could affect sleep and weight status: a population-based study among children

**DOI:** 10.1186/s12966-017-0547-2

**Published:** 2017-07-19

**Authors:** Nomathemba Dube, Kaviul Khan, Sarah Loehr, Yen Chu, Paul Veugelers

**Affiliations:** 1 Department of Public Health Sciences, Population Health Intervention Research Unit, School of Public Health, University of Alberta, 3-50 University Terrace, 8303 112 Street, Edmonton, AB T6G 2T4 Canada; 2 Dalla Lana School of Public Health, University of Toronto, Toronto, ON Canada; 3 Centre for Clinical Epidemiology and Evaluation, University of British Columbia, Vancouver, BC Canada

**Keywords:** Sleep duration, Total time in bed, Sleep quality sleep efficiency, Weight status, Elementary school children, Electronic devices

## Abstract

**Background:**

Short sleep duration and poor sleep quality have been demonstrated to be associated with childhood obesity. It has been suggested that electronic entertainment and communication devices (EECDs) including TVs, computers, tablets, video games and cell phones interfere with sleep in children and youth. The aim of this study was to assess the impact that the use of EECDs in the hour before bedtime has on sleep and weight status to inform sleep promotion interventions and programs to prevent childhood obesity.

**Methods:**

A provincially representative sample of 2334 grade 5 children and their parents in Alberta, Canada was surveyed. Parents reported their child’s bedtime and wake-up time along with how often their child snored, felt sleepy during the day, woke-up at night and woke-up in the morning feeling unrefreshed. Sleep duration, sleep quality and sleep efficiency were derived from these indicators. Parents also reported on the presence of EECDs in their child’s bedroom, while children reported use of EECDs during the day and frequency of using each of these devices during the hour before sleep. The height and weight of children were measured. Multivariable mixed effect linear and logistic regression models were used to determine how sleep duration, sleep quality, sleep efficiency and weight status are influenced by (i) access to EECDs in children’s bedrooms, (ii) use of EECDs during the hour before sleep, and (iii) calming activities specifically reading during the hour before sleep.

**Results:**

Sleep duration was shorter by −10.8 min (cell phone), −10.2 min (computer) and −7.8 min (TV) for those with bedroom access to and used these EECDs during the hour before sleep compared to no access and no use. Good sleep quality was hindered by bedroom access to and use of all EECDs investigated during the hour before sleep, particularly among users of cell phones (OR = 0.64, 95% CI: 0.58–0.71) and computers (OR = 0.72, 95% CI: 0.65–0.80). Very good sleep efficiency was decreased by access to and frequent use of a TV (54%), cell phone (52%), tablet (51%) and video games (51%). Odds of obesity were doubled by bedroom access to and use of a TV and computer during the hour before sleep. Children who rarely read a printed book in the bedroom during the hour before sleep had a shorter sleep duration and poorer sleep quality and sleep efficiency compared to their peers. Having access to an EECD in the bedroom was associated with increased obesity despite frequently reading during the hour before sleep.

**Conclusions:**

Our findings suggest that sleep duration, sleep quality, sleep efficiency and weight status are better among children who do not have EECDs in the bedroom and frequently read a book during the hour before sleep as opposed to those who use EECDs during this hour. Education of limits against EECD use by parents may improve sleep outcomes. These findings will inform health promotion messages and may give rise to national recommendations regarding EECD use.

**Trial registration:**

ClinicalTrials.gov NCT01914185. Registered 31 July 2013 Retrospectively registered.

## Background

The National Sleep Foundation recommends 9–11 h of sleep per night for children aged 6–13 years [1]. Obtaining the recommended amount of sleep is important for mental and physical health [2, 3]. Despite this, children nowadays are sleeping much less than children 20 years ago [4]. Between 1974 and 1986 alone, sleep duration in children was found to have decreased by one hour [4]. The rising use of electronic entertainment and communication devices (EECDs) by children have been shown to play a role in shorter sleep duration [5] as well as poor sleep quality [6] and sleep efficiency [7]. Between 1996 and 2009, children aged 8–18 years old were reported to use EECDs (TVs, computers, tablets, video games and cell phones) for as much as 8 ½ hours daily, seven days a week [8, 9]. The negative effects of long periods of EECD use on sleep are well established [7, 9–11] and so are the effects of sleep deprivation on body weight [10, 12–14], demonstrated by the activation of a hormonal response when sleep is insufficient, which increases appetite and food consumption leading to obesity [15–19]. In an era of deteriorating sleep habits and rising childhood obesity rates, understanding fully which practices affect both sleep duration and sleep quality is essential to public health decision makers.

While access to and use of EECDs in the bedroom have been shown to shorten sleep duration [9], it remains unclear whether it is merely the presence of EECDs in the bedroom, or whether it is their use immediately before bedtime that is detrimental to sleep. It is therefore important to investigate how the presence and use of EECDs in the bedroom affect sleep, and specifically, how sleep may be affected when these devices are used during the hour before bedtime. In the present study, we have investigated the effects of independent and interdependent presence and use of EECDs by grade 5 children in the bedroom during the hour before sleep on: i) sleep duration, ii) sleep quality iii) sleep efficiency and iv) weight status. Furthermore, since reading a printed book has been recommended as a calming activity to help the body wind down and shift into sleep mode [20], we also investigated how reading during the hour before sleep is associated with these four outcomes. Reading a printed book was of interest as opposed to a self-luminous eReader because calming effects of eReaders require further validation [21].

## Methods

### Study population

In 2012, 181 geographically representative elementary schools in Alberta, Canada were invited to participate in the Raising healthy Eating and Active Living Kids in Alberta (REAL Kids Alberta) survey. Of those invited, 143 (77.9%) schools agreed to participate. Packets containing a consent form and a home-based questionnaire [22], both to be completed by parents, were sent home with 4957 grade 5 children, who typically are either 10 or 11 years old. Of the packets sent home, 2732 were returned (55.1% return rate) with 50.1% (2483/4957) of children receiving parental consent. Of the children with parental consent, 94.0% (2334/2483) completed the school-based questionnaire and had heights and weights measured by evaluation assistants while the remaining 6.0% (149/2483) of children with parental consent were either absent on the day of the survey or declined to participate. More information about study tools is available at http://www.REALKidsAlberta.ca.

### Outcomes of interest

#### Sleep duration and Total time in bed

Parents were asked to report their child’s usual bedtime, additional time required to fall asleep after going to bed and wakeup time for weekdays and weekend days. Sleep duration was calculated by subtracting the child’s bedtime and additional time required to fall asleep from wakeup time. Total time in bed (TTIB) was calculated by subtracting the child’s bedtime from wakeup time. For each child, sleep duration across the whole week was calculated by multiplying the weekday sleep duration by five and the weekend sleep duration by two before obtaining the sum of both and dividing it by seven. The same was done for TTIB using weekday and weekend TTIB in bed.

#### Sleep quality and sleep efficiency

Parents were asked four questions about their child’s sleep quality. They were asked whether their child (i) snored; (ii) woke up feeling unrefreshed; (iii) felt sleepy during the daytime; and (iv) woke up during the night after falling asleep. Response options were ‘Never’, ‘Sometimes’, ‘Frequently’ and ‘Almost always’. These sleep quality questions were adapted from validated questions among adults and were used in our population. To obtain sleep efficiency, sleep duration was divided by TTIB, before multiplying the result by 100. Sleep efficiency, was categorized into good (90–94%) and very good (≥95%).

#### Weight status

Body Mass Index (BMI) of each student was calculated using her or his measured height and weight. Children were asked to remove their shoes before the measurements were taken. Height was measured to the nearest 0.1 cm on a portable stadiometer, and body weight was measured to the nearest 0.1 kg on calibrated digital scales. The resulting BMI was used to define weight status based on the age- and gender-specific cut-off points specified by the Extended International Obesity Task Force [23, 24].

### Exposures of interest

#### Access to EECDs in the bedroom

Parents were asked to indicate, which EECDs (TV, computer, tablet, video game or cell phone) their child had access to in the bedroom. Responses included: ‘Yes’, ‘No’ and ‘Sometimes’, where ‘Sometimes’ was considered as ‘Yes’ in the analysis. Additionally, in the analysis, children were considered to have access to “at least one device” in their bedroom if parents responded ‘Yes’ or ‘Sometimes’ to having access to any one of the EECDs in the bedroom.

#### Bedroom use of EECDs during the hour before sleep

Children were asked how often they used each EECD in their bedroom during the hour before sleep. Response options were ‘Never’, ‘About once per month’, ‘1–2 times a week’, ‘3–4 times a week’ and ‘5 or more times per week’. Children whose responses were ‘Never’ or ‘About once per month’ were regarded as ‘Never’ using the EECDs during the hour before sleep, while children providing alternative responses were regarded as ‘Used’ EECDs during that hour.

#### Frequency of reading a book one hour before sleep

Children were asked how often they read in their bedroom during the hour before sleep. Response options were categorized as ‘Rarely’ and ‘Frequently’.

### Other covariates

Parents who completed the survey were asked about the gender of their child, their highest level of educational attainment (secondary or less, college, university or above) and household income levels in Canadian dollars (CA$) (≤CA$50,000; CA$50,001–CA$100,000; or ≥CA$100,000). Region of elementary school (metropolitan, city or rural) was determined using school postal codes. Children were asked how many hours per day they spend (i) using the computer, (ii) playing video games and (iii) watching TV outside of school hours. Response options were ‘Less than 1 hour a day’ regarded as 0.5 h; ‘1 – 2 hours a day’ regarded as 1.5 h; ‘3 – 4 hours a day’ regarded as 3.5 h; and 5 or more hours a day’ regarded as 5.5 h. The total sum of hours each student spent on EECDs was calculated to represent total daily exposure to EECDs.

### Statistical analysis

Sleep quality was derived by using exploratory factor analysis with varimax rotations. The resulting factor scores were grouped into tertiles, with the first representing good sleep quality and the second and third tertiles representing poor sleep quality [9]. For descriptive statistics, one-way analysis of variance (ANOVA) was used to test for differences in the means of sleep duration, a normally distributed continuous variable, and exposure variables. Associations between sleep quality, sleep efficiency and weight status with the exposures of interest were evaluated with Rao-Scott chi-square statistics [25, 26]. When one or more of the variable frequencies was five or less, the Fishers Exact test was used instead. To accommodate clustering of student observations within schools, the effect of EECD access and use during the hour before sleep was evaluated using mixed effect linear regression for sleep duration and TTIB and mixed effect logistic regression for sleep quality and sleep efficiency. For weight status, thinness weight categories (grade 1, grade 2 and grade 3) were combined into one category (underweight) and obese and morbid obese were also combined to form the obese weight category. Multinomial logistic regression was used to evaluate the association between weight status categories (normal, overweight and obese) and the exposure groups. All multivariable analyses were adjusted for gender, household income, parental education, and area of residence as potential confounders. For all regression analyses, total daily exposure to EECDs outside of school hours was adjusted for and a combined effect of total daily exposure to EECDs and exposure during the hour before sleep was computed using linear combinations of estimators. Statistical analyses were performed in STATA version 14 (StataCorp, College Station, Texas). A *p* value of less than 0.05 (two-sided test) was considered statistically significant. Study ethical approval was obtained from University of Alberta Health Research Ethics Board.

## Results

Demographic characteristics of the 2334 children who participated in the survey are shown in Table 1. Sleep duration on weekdays ranged between 7.33 h and 12.58 h, whilst it ranged between 7.00 h and 13.25 h on the weekend. TTIB ranged between 7.50 h and 13.00 h on weekdays and 7.50 h and 14.00 h on the weekend. On average, TTIB were statistically significantly longer for girls than boys. Compared to their peers, longer sleep duration and TTIB were observed amongst children in schools located away from metropolitan areas, who were from high-income families, who were of normal weight or less and were exposed to EECDs for less than two hours a day, (p _trend_ > 0.001) (Table 1).

**Table 1:** Sleep duration and Total time in bed of grade 5 children by gender, highest level of parental education, school region, household income, weight status, total daily exposure to devices and days of the week, Alberta, 2012

Variables	Frequency	(%)	Sleep duration (Hours)	*p-value*	Total Time in Bed (Hours)	*p-value*
Gender						
Girls	1235	(53.2)	9.85 (±0.56)	*0.05*	10.22 (±0.56)	*0.01*
Boys	1071	(46.8)	9.82 (±0.58)		10.16 (±0.59)	
Highest level of parental education						
No school/Elementary school	63	(3.0)	9.83 (±0.58)	0.65	10.19 (±0.58)	0.29
Secondary/college	1391	(59.0)	9.86 (±0.54)		10.21 (±0.54)	
University	792	(38.0)	9.83 (±0.58)		10.17 (±0.59)	
Region of elementary school						
Metropolitan	612	(49.0)	9.78 (±0.59)	*<0.001*	10.11 (±0.60)	*<0.001*
City	781	(16.3)	9.90 (±0.57)		10.21 (±0.54)	
Rural/town	941	(34.7)	9.88 (±0.54)		10.24 (±0.58)	
Household income						
< $50,000 (Low)	385	(19.7)	9.71 (±0.58)	*<0.001*	10.08 (±0.57)	*<0.001*
$50,001 - $100,000 (Middle)	614	(26.6)	9.84 (±0.55)		10.21 (±0.55)	
> $100,000 (High)	684	(28.2)	9.90 (±0.55)		10.22 (±0.56)	
Don’t know/Prefer not to answer*	575	(25.5)	9.91 (±0.57)		10.22 (±0.59)	
Weight category						
Thinness grade 3	20	(0.9)	10.06 (±0.63)	** < 0.001	10.38 (±0.63)	*<0.001*
Thinness grade 2	49	(2.1)	9.91 (±0.71)		10.27 (±0.71)	
Thinness grade 1	233	(10.1)	9.98 (±0.51)		10.30 (±0.51)	
Normal	1503	(65.0)	9.88 (±0.57)		10.21 (±0.61)	
Overweight	399	(17.3)	9.75 (±0.60)		10.08 (±0.57)	
Obese	48	(2.1)	9.69 (±0.57)		10.01 (±0.01)	
Morbid Obese	3	(0.1)	9.80 (±0.14)		10.20 (±0.59)	
Total daily exposure to devices (hours)						
< 2	438	(18.8)	9.97(±0.55)	*<0.001*	10.29 (±0.56)	*<0.001*
2.0	1045	(44.1)	9.89 (±0.52)		10.22 (±0.53)	
3.5	48	(2.2)	9.71(±0.54)		10.04 (±0.51)	
4.0	264	(12.1)	9.82 (±0.62)		10.14 (±0.61)	
5.0	201	(9.5)	9.72 (±0.54)		10.07 (±0.66)	
5.5	318	(13.3)	9.78 (±0.60)		10.12 (±0.61)	
*Days of the week*						
Week days	2276	(97.5)	9.78 (±0.64)	*<0.001*	10.12 (±0.63)	*<0.001*
Weekend days	2.263	(97.0)	9.99 (±0.72)		10.37 (±0.79)	

*Household income category “Don’t know/Prefer not to answer” excluded from p trend calculations.

**p values generated using an aggregate of ‘thinness grade 1’, thinness grade 2′ and thinness grade 3′ and ‘obese and morbid obese’ categories

*p* value <0.05 was considered statistically significant

Good sleep quality was more likely to be observed among children whose parents had a university education (p _trend_ < 0.001) and who were from high-income families (p _trend_ < 0.001) (Table 2). Average very good sleep efficiency was 97.6% ± 1.2% (range: 95.0% – 99.3%).

**Table 2:** Sleep quality and sleep efficiency of grade 5 children by gender, highest level of parental education, school region, household income, weight status and total daily exposure to devices, Alberta, 2012

Variables	Frequency	(%)	Good sleep quality (%)	*p-value*	Very good sleep efficiency (%)	*p-value*
Gender						
Girls	1235	(53.2)	32.5	0.08	94.2	0.96
Boys	1071	(46.8)	37.1		94.2	
Highest level of parental education						
No school/Elementary school	63	(3.0)	30.3	*<0.001*	92.2	0.18
Secondary/college	1391	(59.0)	32.6		94.4	
University	792	(38.0)	39.8		95.2	
Region of elementary school						
Metropolitan	612	(49.0)	36.5	0.30	94.7	0.63
City	781	(16.3)	32.9		94.1	
Rural/town	941	(34.7)	33.7		93.5	
Household income						
< $50,000 (Low)	385	(19.7)	22.9	*<0.001*	94.0	0.60
$50,001 - $100,000 (Middle)	614	(26.6)	32.7		94.4	
> $100,000 (High)	684	(28.2)	35.1		94.2	
Don’t know/Prefer not to answer*	575	(25.5)	38.4		94.7	
Weight category**						
Thinness grade 3	20	(0.9)	21.5	***0.10	100.0	***0.53
Thinness grade 2	49	(2.1)	38.0		89.6	
Thinness grade 1	233	(10.1)	35.9		94.5	
Normal	1503	(65.0)	36.2		94.9	
Overweight	399	(17.3)	29.8		92.8	
Obese	48	(2.1)	22.5		94.3	
Morbid Obese	3	(0.1)	26.6		100.0	
Total daily exposure to devices (hours)						
< 2	438	(18.8)	37.4	0.07	96.4	0.06
2.0	1045	(44.1)	36.8		94.5	
3.5	48	(2.2)	33.9		89.5	
4.0	264	(12.1)	32.7		93.8	
5.0	201	(9.5)	28.4		92.5	
5.5	318	(13.3)	30.4		92.5	

*Household income category “Don’t know/Prefer not to answer” excluded from p trend calculations

**Missing weight category excluded

***p values generated using an aggregate of ‘thinness grade 1’, thinness grade 2′ and thinness grade 3′ and ‘obese and morbid obese’ categories

*p* value <0.05 was considered statistically significant

Sleep duration and TTIB were statistically significantly shorter for those with access to a TV, computer, video game and cell phone in the bedroom compared to those without (Table 3). Frequently reading a book in the bedroom with no access to EECDs was associated with longer sleep duration, longer TTIB (Table 3), the highest sleep quality and better sleep efficiency (Table 4) compared to other reading categories.

**Table 3:** Sleep duration and Total time in bed of grade 5 children and electronic and entertainment communication device bedroom access and use during the hour before sleep

	%	Sleep duration (Hours)	*p-value*	Total Time in Bed (%)	*p-value*
TV					
No access and no use	53.7	9.87 (±0.56)	*<0.001*	10.19 (±0.56)	*<0.001*
Access but never used	14.2	9.80 (±0.59)		10.12 (±0.61)	
Access and used	20.1	9.75 (±0.60)		10.09 (±0.60)	
^a^No access and used	12.0	9.90 (±0.54)		10.23 (±0.54)	
Computer					
No access and no use	54.5	9.89 (±0.56)	*<0.001*	10.21 (±0.57)	*<0.001*
Access but never used	13.8	9.77 (±0.59)		10.10 (±0.60)	
Access and used	12.3	9.70 (±0.63)		10.02 (±0.64)	
^a^No access and used	19.4	9.84 (±0.54)		10.17 (±0.54)	
Tablet					
No access and no use	52.3	9.86 (±0.57)	0.50	10.18 (±0.58)	0.52
Access but never used	10.2	9.83 (±0.65)		10.15 (±0.65)	
Access and used	16.3	9.83 (±0.56)		10.17 (±0.56)	
^a^No access and used	21.2	9.83 (±0.55)		10.15 (±0.56)	
Video Games					
No access and no use	60.9	9.87 (±0.55)	*<0.001*	10.19 (±0.56)	*0.002*
Access but never used	15.9	9.78 (±0.58)		10.11 (±0.58)	
Access and used	10.3	9.78 (±0.65)		10.12 (±0.65)	
^a^No access and used	12.9	9.85 (±0.62)		10.19 (±0.60)	
Cell Phone					
No access and no use	59.0	9.89 (±0.57)	*<0.001*	10.21 (±0.58)	*<0.001*
Access but never used	23.1	9.78 (±0.53)		10.11 (±0.53)	
Access and used	11.8	9.73 (±0.57)		10.06 (±0.57)	
^a^No access and used	6.1	9.87 (±0.69)		10.19 (±0.69)	
At least one device					
No access and no use	12.2	9.93 (±0.56)	*<0.001*	10.27 (±0.57)	*<0.001*
Access but never used	17.8	9.82 (±0.58)		10.15 (±0.60)	
Access and used	56.4	9.80 (±0.57)		10.13 (±0.58)	
^a^No access and used	13.6	9.96 (±0.53)		10.26 (±0.54)	
Reading					
No access and frequent reading	19.9	9.97 (±0.53)	*<0.001*	10.28 (±0.54)	*<0.001*
Access but frequent reading	52.9	9.83 (±0.58)		10.16 (±0.59)	
Access and rarely reading	21.2	9.74 (±0.56)		10.08 (±0.56)	
No access and rarely reading	6.0	9.89 (±0.60)		10.19 (±0.60)	

^a^Response category considered illogical: No access to EECD in the bedroom and is used in the bedroom during the hour before sleep

*p* value <0.05 was considered statistically significant

**Table 4:** Sleep quality and sleep efficiency of grade 5 children and electronic and entertainment communication device bedroom access and use during the hour before sleep

	%	Good sleep quality (%)	*p-value*	Very good sleep efficiency (%)	*p-value*
TV					
No access and no use	53.7	36.3	0.67	94.5	*0.01*
Access but never used	14.2	35.9		96.4	
Access and used	20.1	32.1		90.8	
^a^No access and used	12.0	32.4		94.7	
Computer					
No access and no use	54.5	36.4	0.17	94.5	0.71
Access but never used	13.8	31.5		93.1	
Access and used	12.3	30.1		93.1	
^a^No access and used	19.4	36.2		93.5	
Tablet					
No access and no use	52.3	36.2	0.67	94.3	0.64
Access but never used	10.2	32.7		93.7	
Access and used	16.3	35.0		92.5	
^a^No access and used	21.2	33.5		94.3	
Video Games					
No access and no use	60.9	36.3	0.77	95.1	*0.01*
Access but never used	15.9	31.6		94.6	
Access and used	10.3	34.2		91.9	
^a^No access and used	12.9	35.0		90.6	
Cell Phone					
No access and no use	59.0	36.6	0.10	95.1	*0.03*
Access but never used	23.1	33.2		93.1	
Access and used	11.8	28.9		90.6	
^a^No access and used	6.1	38.8		94.6	
At least one device					
No access and no use	12.2	35.5	0.29	94.9	0.06
Access but never used	17.8	36.9		95.7	
Access and used	56.4	33.1		92.8	
^a^No access and used	13.6	39.1		96.0	
Reading					
No access and frequent reading	19.9	38.4	*<0.001*	96.3	*0.01*
Access but frequent reading	52.9	37.5		94.5	
Access and rarely reading	21.2	25.9		91.0	
No access and rarely reading	6.0	34.1		92.7	

^a^Response category considered illogical: No access to EECD in the bedroom and is used in the bedroom during the hour before sleep

*p* value <0.05 was considered statistically significant

Furthermore, an increase in the proportion of children with good sleep quality (additional 2.1%) was observed amongst those who both frequently read during the hour before sleep and had no EECDs accessible in the bedroom (Data not shown).

When compared to normal weight children, a larger proportion of obese children had access to EECDs in the bedroom and used them during the hour before sleep, except for the tablet, which was used by a large proportion of normal weight children. However, a statistically significant difference in proportion of weight status was only observed with the TV (Table 5).

**Table 5:** Weight status characteristics of grade 5 children and electronic and entertainment communication device bedroom access and use during the hour before sleep

	%	^a^Underweight (13.4%) %	Normal (66.7%) %	Overweight (17.7%) %	^b^Obese (2.3%) %	*p-value*
*TV*						
No access and no use	53.7	61.7	54.0	49.7	28.6	*<0.001*
Access but never used	14.2	8.5	14.2	16.5	22.5	
Access and used	20.1	15.3	19.9	24.6	38.8	
^c^No access and used	12.0	14.6	11.9	9.2	10.2	
Computer						
No access and no use	54.5	55.5	57.1	57.0	43.8	0.23
Access but never used	13.8	12.3	13.8	12.7	16.7	
Access and used	12.3	10.3	10.2	12.1	22.9	
^c^No access and used	19.4	21.9	18.9	18.2	16.7	
*Tablet*						
No access and no use	52.3	50.7	50.7	54.4	50.0	0.82
Access but never used	10.2	9.3	9.9	8.8	8.7	
Access and used	16.3	16.4	17.7	13.6	15.2	
^c^No access and used	21.2	23.6	21.7	23.2	26.1	
Video Games						
No access and no use	60.9	64.1	60.3	62.6	52.1	0.17
Access but never used	15.9	12.1	15.9	16.3	27.1	
Access and used	10.3	9.3	10.4	10.3	14.6	
^c^No access and used	12.9	14.5	13.4	10.8	6.3	
Cell Phone						
No access and no use	59.0	56.7	61.1	55.6	53.1	0.39
Access but never used	23.1	25.9	21.5	26.6	24.5	
Access and used	11.8	10.2	11.9	12.3	14.3	
^c^No access and used	6.1	7.2	5.6	5.5	8.2	
At least one device						
No access and no use	12.2	12.8	12.3	12.1	6.0	0.56
Access but never used	17.8	17.2	17.9	17.7	8.0	
Access and used	56.4	54.7	56.3	57.7	72.0	
^c^No access and used	13.6	15.2	13.5	12.7	14.0	
Reading						
No access and frequent reading	19.9	21.6	20.1	16.8	14.0	0.66
Access but frequent reading	52.9	49.7	53.1	52.2	56.0	
Access and rarely reading	21.2	22.3	21.0	23.3	24.0	
No access and rarely reading	6.0	6.4	5.8	7.8	6.0	

^a^Thinness grade 1, Thinness grade 2 and Thinness grade 3 weight categories combined

^b^Obese and Morbid obese weight categories combined

^c^Response category considered illogical: No access to EECD in the bedroom and is used in the bedroom during the hour before sleep

*p* value <0.05 was considered statistically significant

### Effects of EECD access and use on sleep duration and Total time in bed

After adjusting for covariates, children with access to EECDs in the bedroom and reportedly used them during the hour before sleep reported shorter sleep duration of −10.8 min for a cell phone (β = −0.18 h; 95% CI = −0.26, −0.09), −10.2 min for a computer (β = −0.17 h; 95% CI = −0.26, −0.08) and −7.8 min for a TV (β = −0.13 h; 95% CI = −0.20, −0.06) compared to the reference (Table 6). These findings coincided with a reduced TTIB of −7.8 min for a cell phone (β = −0.13 h; 95% CI = −0.22, −0.05), −9.0 min for a computer (β = −0.15 h; 95% CI = −0.23, −0.06) and −4.2 min for TV (β = −0.07 h; 95% CI = −0.14, −0.00) among children with access to EECDs in the bedroom and reportedly used them during the hour before sleep. Interestingly, when a child had access to a cell phone in the bedroom, their sleep duration decreased (−7.8 min) as well as their TTIB (−5.4 min) (Table 6). Access to and frequent use of tablets and video games in the bedroom during the hour before sleep had no statistically significant effect on sleep duration and TTIB.

Among children who frequently read during the hour before sleep, those with an EECD present in the bedroom had a shorter sleep duration of −9.0 min (β = −0.15 h; 95% CI = −0.23, −0.08) and a shorter TTIB of −6.6 min (β = −0.11 h; 95% CI = −0.18, −0.05) compared to those without an EECD in the bedroom. Rarely reading with an EECD present in the bedroom shortened sleep duration (−12.6 min) and TTIB (−9.6 min) further (Table 6).

**Table 6:** Effect of electronic entertainment and communication device use during the day and access and use in the bedroom during the hour before sleep on Sleep duration and Total time in bed in grade 5 children

	Sleep duration		Total time in bed	
	Unadjusted coefficient (95% CI)	^a^Adjusted coefficient (95% CI)	Unadjusted coefficient (95% CI)	^a^Adjusted coefficient (95% CI)
TV				
No access and no use	^b^Ref	Ref	Ref	Ref
Access but never used	−0.07 (−0.16, 0.02)	*−0.09 (−0.18, −0.01)*	−0.07 (−0.16, 0.02)	−0.07 (−0.15, 0.02)
Access and frequently used	*−0.10 (−0.17, −0.03)*	*−0.13 (−0.20, −0.06)*	*−0.08 (−0.15, −0.00)*	*−0.07 (−0.14, −0.00)*
Computer				
No access and no use	Ref	Ref	Ref	Ref
Access but never used	−0.05 (−0.17, 0.06)	−0.12 (−0.22, 0.02)	−0.10 (−0.20, 0.00)	−0.09 (−0.19, 0.01)
Access and frequently used	*−0.14 (−0.24, −0.05)*	*−0.17 (−0.26, −0.08)*	*−0.12 (−0.22, −0.02)*	*−0.15 (−0.23, −0.06)*
Tablet				
No access and no use	Ref	Ref	Ref	Ref
Access but never used	−0.03 (−0.15, 0.08)	−0.08 (−0.19, 0.03)	−0.05 (−0.13, 0.03)	−0.04 (−0.11, 0.03)
Access and frequently used	−0.05 (−0.15, 0.05)	−0.09 (−0.18, 0.01)	−0.05 (−0.15, 0.05)	−0.01 (−0.11, 0.07)
Video Games				
No access and no use	Ref	Ref	Ref	Ref
Access but never used	−0.06 (−0.14, 0.02)	*−0.08 (−0.16, −0.01)*	−0.02 (−0.13, 0.09)	−0.04 (−0.14, 0.07)
Access and frequently used	−0.07 (−0.18, 0.03)	−0.08 (−0.17, 0.02)	−0.03 (−0.13, 0.06)	−0.03 (−0.13, 0.06)
Cell Phone				
No access and no use	Ref	Ref	Ref	Ref
Access but never used	*−0.12 (−0.18, −0.05)*	*−0.13 (−0.20, −0.07)*	*−0.12 (−0.18, −0.05)*	*−0.09 (−0.15, −0.03)*
Access and frequently used	*−0.16 (−0.24, −0.08)*	*−0.18 (−0.26, −0.09)*	*−0.16 (−0.24, −0.08)*	*−0.13 (−0.22, −0.05)*
At least one device				
No access and no use	Ref	Ref	Ref	Ref
Access but never used	−0.08 (−0.20, 0.04)	−0.11 (−0.24, 0.03)	−0.08 (−0.20, 0.05)	−0.07 (−0.21, 0.06)
Access and frequently used	*−0.11 (−0.20, −0.02)*	*−0.13 (−0.23, −0.03)*	*−0.11 (−0.20, −0.02)*	*−0.10 (−0.20, −0.01)*
Reading				
No access and frequent reading	Ref	Ref	Ref	Ref
Access but frequent reading	*−0.12 (−0.19, −0.05)*	*−0.15 (−0.23, −0.08)*	*−0.11 (−0.18, −0.05)*	*−0.11 (−0.18, −0.05)*
Access and rarely reading	*−0.19 (−0.27, −0.11)*	*−0.21 (−0.30, −0.13)*	*−0.17 (−0.25, −0.09)*	*−0.16 (−0.24, −0.07)*
No access and rarely reading	−0.04 (−0.17, 0.08)	−0.07 (−0.19, 0.06)	−0.06 (−0.18, 0.06)	−0.05 (−0.18, 0.07)

^a^Adjusted for gender of the child, household income, region of elementary school, highest level of parental education and total daily exposure to electronic entertainment and communication devices

^b^Reference category

Daily exposure to EECDs was associated with a decrease in sleep duration of between −2.1 and −2.2 min for all EECDs. A similar association was observed among children who used EECDs during the day and read during the hour before sleep (Data not shown).

### Effects of EECD access and use on sleep quality

After adjusting for covariates, access to a computer, tablet, video games or cell phone in the bedroom, whether used or not during the hour before sleep (compared to the reference) decreased the odds of good sleep quality (Table 7). Having a TV in the bedroom affected good sleep quality when it was frequently used during the hour before sleep (OR = 0.86, 95% CI: 0.80, 0.94) (Table 7). Cell phone access and its frequent use during the hour before sleep had the greatest impact on good sleep quality, decreasing it by 36% (OR = 0.64, 95% CI: 0.58, 0.71) (Table 7).

**Table 7:** Effect of electronic entertainment and communication device use during the day and access and use in the bedroom during the hour before sleep on sleep quality and sleep efficiency in grade 5 children

	Good sleep quality		Very good sleep efficiency (≥95%)	
	Unadjusted OR (95% CI)	^a^Adjusted OR (95% CI)	Unadjusted OR (95% CI)	^a^Adjusted OR (95% CI)
TV				
No access and no use	^b^Ref	Ref	Ref	Ref
Access but never used	*1.09 (1.01, 1.19)*	1.01 (0.93, 1.11)	*1.93 (1.56, 2.39)*	*1.45 (1.13, 1.85)*
Access and frequently used	*0.90 (0.84, 0.97)*	*0.86 (0.80, 0.94)*	*0.58 (0.50, 0.66)*	*0.46 (0.39, 0.56)*
Computer				
No access and no use	Ref	Ref	Ref	Ref
Access but never used	*0.82 (0.75, 0.90)*	*0.73 (0.67, 0.80)*	*0.77 (0.65, 0.91)*	*0.83 (0.69, 0.98)*
Access and frequently used	*0.83 (0.75, 0.91)*	*0.72 (0.65, 0.80)*	1.19 (0.98, 1.44)	0.96 (0.79, 1.18)
Tablet				
No access and no use	Ref	Ref	Ref	Ref
Access but never used	*0.84 (0.76, 0.93)*	*0.79 (0.71, 0.87)*	0.97 (0.81, 1.15)	*0.58 (0.48, 0.71)*
Access and frequently used	*0.87 (0.81, 0.95)*	*0.82 (0.76, 0.89)*	*0.55 (0.46, 0.65)*	*0.49 (0.42, 0.58)*
Video Games				
No access and no use	Ref	Ref	Ref	Ref
Access but never used	*0.86 (0.79, 0.93)*	*0.80 (0.74, 0.87)*	*0.74 (0.61, 0.89)*	0.91 (0.76, 1.10)
Access and frequently used	0.99 (0.90, 1.09)	*0.87 (0.79, 0.97)*	*0.55 (0.48, 0.64)*	*0.49 (0.41, 0.59)*
Cell Phone				
No access and no use	Ref	Ref	Ref	Ref
Access but never used	*0.84 (0.78, 0.90)*	*0.73 (0.69, 0.80)*	*0.84 (0.78, 0.90)*	*0.56 (0.49, 0.65)*
Access and frequently used	*0.67 (0.61, 0.74)*	*0.64 (0.58, 0.71)*	*0.67 (0.61, 0.74)*	*0.48 (0.41, 0.57)*
At least one device				
No access and no use	Ref	Ref	Ref	Ref
Access but never used	1.10 (0.99, 1.22)	0.97 (0.87, 1.08)	*1.29 (1.01 1.65)*	1.06 (0.82, 1.37)
Access and frequently used	0.96 (0.88, 1.05)	*0.87 (0.79, 0.95)*	*0.59 (0.48, 0.72)*	*0.52 (0.42, 0.63)*
Reading				
No access and frequent reading	Ref	Ref	Ref	Ref
Access but frequent reading	0.99 (0.92, 1.06)	*0.87 (0.81, 0.94)*	*0.62 (0.52, 0.75)*	*0.66 (0.55, 0.80)*
Access and rarely reading	*0.61 (0.56, 0.67)*	*0.57 (0.52, 0.63)*	*0.36 (0.29, 0.43)*	*0.33 (0.27, 0.40)*
No access and rarely reading	0.95 (0.83, 1.08)	*0.86 (0.76, 0.99)*	*0.57 (0.44, 0.76)*	0.77 (0.58, 1.03)

^a^Adjusted for gender of the child, household income, region of elementary school, highest level of parental education and total daily exposure to electronic entertainment and communication devices

^b^Reference category

Having access to any EECD and rarely reading during the hour before sleep (compared to no access and frequently reading) decrease good sleep quality by 43% (OR = 0.57, 95% CI: 0.52, 0.63) (Table 7). Among children with access to EECDs in the bedroom, those who rarely read (compared to those who frequently read) had a 34% decrease in good sleep quality (OR = 0.66, 95% CI: 0.61, 0.71) (Data not shown).

Total daily exposure to EECDs was consistently associated with lower good sleep quality of between 8% and 9% for all EECDs investigated. Exposure to EECDs during the day also decreased good sleep quality among children who frequently read during the hour before sleep (Data not shown).

### Effects of EECD access and use on sleep efficiency

Although frequently using the TV during the hour before sleep significantly decreased very good sleep efficiency by 54% (OR = 0.46, 95% CI: 0.39, 0.56), it was notable that, the odds of very good sleep efficiency were increased by 45% among children with access to a TV in the bedroom, though not used during the hour before sleep (OR = 1.45, 95% CI: 1.13, 1.85) (Table 7). The effect of having access to a tablet or cell phone in the bedroom, whether used or not during the hour before sleep, affected very good sleep efficiency to a similar extent (Table 7). Never using a computer during the hour before sleep, although accessible in the bedroom, decreased very good sleep efficiency by 17% (OR = 0.83, 95% CI: 0.69, 0.98) (Table 7).

Compared to having no access to EECDs and frequently reading during the hour before sleep, access to EECDs in the bedroom, whether one frequently read during the hour before sleep (OR = 0.66, 95% CI: 0.55, 0.80) or not (OR = 0.33, 95% CI: 0.27, 0.40), was associated with a 34% and 67% decrease in very good sleep efficiency respectively. Among children with access to EECDs in the bedroom, those who rarely read (compared to those who frequently read) had a 50% decrease in very good sleep efficiency (OR = 0.50, 95% CI: 0.33, 0.57) (Data not shown). Among those who rarely read, having no access to EECDs in the bedroom doubled the odds of having very good sleep efficiency (OR = 2.04, 95% CI: 1.57, 2.65) when compared to having access (Data not shown).

### Effects of EECD access and use on weight status

After adjusting for covariates, when compared to the reference, access to a TV and a computer, when frequently used during the hour before sleep increased the odds of being overweight by more than 20% and doubled the odds of being obese (Table 8). When accessible in the bedroom, a cell phone was associated with an increase in odds of being overweight (OR = 1.44, 95% CI: 1.32, 1.58) and obese (OR = 1.56, 95% CI: 1.24, 1.98), despite it never being used during the hour before sleep. Similarly, access to a TV and video games in the bedroom greatly increased the odds of obesity although reportedly not used during the hour before sleep (Table 8). Overall, access to at least one device in the bedroom, and frequently using it was associated with an 82% increase in odds of obesity (OR = 1.82, 95% CI: 1.28, 2.59) (Table 8).

**Table 8:** Effect of electronic entertainment and communication device use during the day and access and use in the bedroom during the hour before sleep on weight status in grade 5 children

	Overweight vs. Normal		Obese vs. Normal	
	Unadjusted OR (95% CI)	^a^Adjusted OR (95% CI)	Unadjusted OR (95% CI)	^a^Adjusted OR (95% CI)
TV				
No access and no use	^b^Ref	Ref	Ref	Ref
Access but never used	*1.20 (1.08, 1.32)*	*1.15 (1.04, 1.28)*	*2.42 (1.86, 3.14)*	*2.28 (1.74, 2.98)*
Access and frequently used	*1.22 (1.11, 1.33)*	*1.21 (1.10, 1.33)*	*2.90 (2.31, 3.64)*	*2.56 (2.02, 3.24)*
Computer				
No access and no use	Ref	Ref	Ref	Ref
Access but never used	1.01 (0.91, 1.13)	0.99 (0.89, 1.11)	1.08 (0.80, 1.46)	1.17 (0.86, 1.59)
Access and frequently used	*1.37 (1.23, 1.53)*	*1.34 (1.20, 1.50)*	*2.28 (1.77, 2.94)*	*2.79 (2.15, 3.63)*
Tablet				
No access and no use	Ref	Ref	Ref	Ref
Access but never used	*0.81 (0.71, 0.91)*	*0.76 (0.66, 0.86)*	0.73 (0.49, 1.07)	0.84 (0.57, 1.25)
Access and frequently used	*0.72 (0.65, 0.80)*	*0.75 (0.67, 0.84)*	1.12 (0.86, 1.46)	1.19 (0.91, 1.57)
Video Games				
No access and no use	Ref	Ref	Ref	Ref
Access but never used	1.03 (0.94, 1.14)	0.99 (0.89, 1.09)	*1.77 (1.40, 2.23)*	*1.61 (1.27, 2.05)*
Access and frequently used	1.02 (0.90, 1.14)	*0.80 (0.70, 0.91)*	1.32 (0.98, 1.78)	1.10 (0.80, 1.50)
Cell Phone				
No access and no use	Ref	Ref	Ref	Ref
Access but never used	*1.41 (1.29, 1.53)*	*1.44 (1.32, 1.58)*	*1.45 (1.15, 1.82)*	*1.56 (1.24, 1.98)*
Access and frequently used	*1.20 (1.08, 1.34)*	*1.28 (1.14, 1.43)*	1.25 (0.93, 1.68)	1.31 (0.96, 1.78)
At least one device				
No access and no use	Ref	Ref	Ref	Ref
Access but never used	0.99 (0.86, 1.12)	0.92 (0.80, 1.05)	*0.55 (0.34, 0.90)*	*0.54 (0.33, 0.88)*
Access and frequently used	*1.13 (1.01, 1.27)*	1.06 (0.94, 1.19)	*2.04 (1.44, 2.89)*	*1.82 (1.28, 2.59*)
Reading				
No access and frequent reading	Ref	Ref	Ref	Ref
Access but frequent reading	*1.22 (1.22, 1.34)*	*1.18 (1.07, 1.31)*	*1.42 (1.09, 1.86)*	*1.35 (1.03, 1.77)*
Access and rarely reading	*1.41 (1.26, 1.58)*	*1.19 (1.06, 1.34)*	*1.43 (1.05, 1.96)*	1.09 (0.79, 1.51)
No access and rarely reading	*1.64 (1.40, 1.92)*	*1.42 (1.20, 1.68)*	1.38 (0.86, 2.19)	1.11 (0.69, 1.79)

^*a*^Adjusted for gender of the child, household income, region of elementary school, highest level of parental education and total daily exposure to electronic entertainment and communication devices

^b^Reference category

Amongst those with no bedroom EECD access, rarely reading during the hour before sleep (compared to frequently reading) increased the odds of being overweight by 42% (OR = 1.42, 95% CI: 1.20, 1.68) (Table 8). In addition, frequently reading with access to EECDs in the bedroom (compared to without) increased the odds of obesity by 35% (Table 8).

Total daily exposure to EECDs was statistically significantly associated with an increase in weight status ranging from 4% to 7% increase in the odds of being overweight and 24% to 28% increase in odds of being obese for all EECDs (Data not shown).

## Discussion

In the present study, we found that for most EECDs, both their presence in the bedroom and their frequent use during the hour before sleep was negatively associated with sleep duration, good sleep quality and very good sleep efficiency and positively associated with weight status. As expected, the magnitude at which sleep and weight status was affected depended on the EECD in question, however, the TV, computer and cell phone were identified as common culprits. Rarely reading during the hour before sleep was associated with a decrease in sleep duration, sleep quality and sleep efficiency and an increase in the chance of being overweight. Having access to EECDs in the bedroom was associated with an increase in being obese even if children read frequently during the hour before sleep. Exposure to EECDs during the day also contributed to their effect on sleep and weight status when they were used in the bedroom during the hour before sleep.

Our findings support the American Academy of Pediatrics recommendation to remove EECDs from the bedroom to improve sleep amongst children [27]. The observed negative effect on sleep and weight status that the presence of EECDs and their use during the hour before sleep have, suggests that an interdependent relationship exists between EECD presence and use in the bedroom during the hour before sleep. This finding begs for restrictions to EECD use during the hour before sleep as well as their accessibility in the bedroom. Some may argue that the decrease in sleep duration associated with the use of EECDs an hour before sleep is modest. On the contrary, as little as 15 min difference in sleep duration has been shown to have clinical significance on mental, behavioral and daytime functioning [28]. Where multiple EECDs are used, this threshold of 15 min will be exceeded as demonstrated in a similar study [9]. Our findings also show an association of sleep and weight status with total exposure to EECDs during the day. Parents should therefore be encouraged to implement EECD limits on use during the daytime in addition to restrictions to bedroom accessibility and use during the hour before sleep. This should begin while their children are young so as to develop habits that can be continued through to the teenage and adult years.

The negative impact of EECDs when used in the bedroom during the hour before sleep may be related to the bright light emitted from many of these devices [16]. The backlight of many EECDs currently being manufactured emits diodes rich in blue light. Exposure to blue light close to bedtime suppresses the release of the sleep-facilitating hormone; melatonin, thereby delaying sleep onset, shortening total sleep duration and affecting good sleep quality [29, 30]. Exposure time needed to significantly suppress melatonin production may however, differ between products depending on their built-in ability to decrease brightness as well as filter out blue light [21]. In an experimental trial involving tablets, researchers found that after exposure to light from self-luminous Apple iPad® tablets for one hour, the tablets’ built-in lighting did not suppress melatonin production significantly, and therefore, did not delay sleep onset. However, after a two-hour exposure to the same tablets, melatonin production was significantly suppressed, delaying sleep onset [21]. The effect of tablets on sleep latency observed in this trial is consistent with the effect of tablets on sleep duration and weight status observed in our study. We suspect that the duration of exposure (1 h or less) amongst children who used tablets before sleep was insufficient to significantly suppress melatonin production and to have a meaningful effect on sleep duration.

A number of cross-sectional studies have shown that EECDs affect sleep and weight status when accessible in the bedroom even if they are reportedly not used [31–34]. Similarly, in our study, a large number of EECDs investigated negatively affected sleep and weight status when accessible in the bedroom even if children reported these were not used during the hour before sleep. A possible reason for this maybe that the presence of EECDs in the bedroom is a mediator for use [35]. Therefore, we speculate that reporting bias regarding actual use of EECDs during the hour before sleep may exist which explains why sleep (duration, quality, and efficiency) as well as weight status was negatively affected when EECDs were accessible in the bedroom, though reportedly never used during the hour before sleep. These children may have used these devices but reported otherwise due to fear of their parents finding out. The positive effect that TV access in the bedroom had on very good sleep efficiency among students who never used it during the hour before sleep is notable. This is inconsistent with the effect that other screens investigated here have shown. Chaput et al. (2014) recently found that the presence of one screen in the bedroom does not lower sleep efficiency. Although number of screens in the bedroom was not investigated here, the presence of only the TV in the bedroom that was never used decreased any sleep interruptions and therefore increased very good sleep efficiency. As opposed to a TV, having access to a computer, tablet, video games and a cell phone though never used an hour before sleep negatively affected very good sleep efficiency. Cell phone access in the bedroom, even when not in use, has previously been shown to impact negatively on sleep by giving off alerts for incoming calls or messages [36]. By the same token, tablets may give off alerts even when not in use while computers and video games may continually flicker and make sounds that disturb sleep, particularly when left in stand-by mode.

The increase in weight status associated with the interdependent presence and use of EECDs during the hour before sleep is consistent with our previous study which reported that both presence of EECDs in the bedroom and their use at night were associated with lower diet quality, less physical activity and increased odds of being overweight or obese in a different sample of grade 5 students [9]. As expected, the use of EECDs at night displaces sleep thereby shortening sleep duration. Typically, short sleep duration impacts weight status by decreasing the production of leptin, an appetite-suppressing hormone, and increasing that of ghrelin, an appetite-stimulating hormone causing those using EECDs closer to bedtime to eat more [37, 38].

The potential for negative long-term impacts of EECD use at night, may indicate an urgency to address the use of EECDs before sleep. Nuutinen et al. (2013) reported that children who watched TV and used a computer in their bedroom had significantly shorter sleep duration and later bedtimes than their counterparts. Shorter sleep duration persisted 18 months later [31], suggesting long-term negative effects of EECD use in the bedroom. In fact, the World Health Organization has classified exposure to bright light after dark as a carcinogen after observations of increased incidence of cancers amongst night-shift workers [39–41]. Prolonged exposure to bright light after dark, such as what occurs among chronic night-users of EECDs, causes chronic suppression of melatonin.

In place of engaging in stimulating interactive activities with bright lights, we found that frequently reading a book during the hour before sleep without EECD access in the bedroom, is key to a longer sleep duration, good sleep quality, very good sleep efficiency, and normal weight status. A recent study showed that reading a printed book under reflected light has a significant positive impact on restorative sleep, morning alertness and the circadian cycle compared to reading a book from a light-emitting eReader [29]. Based on our results and other supporting literature [29, 30], we recommend that children should not have access EECDs in their bedroom, and should refrain from using EECDs during the hour before sleep. Instead, parents should encourage children to read a book, preferably printed and with dim lighting, during the hour before sleep. Parental rules to control chronic EECD use have been found to be effective [42], and may be applied in our population.

Our findings need to be interpreted within confines of a number of limitations. Firstly, as a cross sectional study, temporality, hence, causality cannot be determined. The relationship of the exposures (EECDs access and use) and the outcomes (sleep duration, sleep quality, sleep efficiency and weight status) may be bidirectional [43]. Secondly, all sleep measures are self-reported and based mainly on parental reports. Parental reports regarding sleep problems have been shown to disagree widely with reports from children, and are therefore prone to error [44]. Reports of children as young as 6 years old have been shown to be valid and add valuable information regarding medical history, behavior and health care [45]. Nonetheless, a more objective measure of sleep, such as that from actigraphy, may improve the validity of study findings. Thirdly, the sample, comprising exclusively of grade 5 elementary school children, is not representative of the general population limiting generalizability; however, this does not discredit any associations found. Lastly, the outcomes studied may have been associated with additional factors such as how long the children were exposed to EECDs during the hour before sleep. Such factors not investigated here could have resulted in residual confounding. Further experimental studies should be conducted to explore how EECD content, type of video games or various uses of cell phones (e.g. calling versus social media) affects sleep and weight status. Additionally, experimental studies on how best to prevent EECD use during the hour before sleep in an era of booming technology and promote reading a printed book instead are required. Despite these limitations, our study has several strengths. As a school-based survey, the response rate and resulting large sample size increased our study power. This is the first study, to our knowledge, to specifically focus on the use of a wide range of EECDs and distinguish between mere bedroom access and actual use within a specified time period before sleep, and how this impacts sleep and weight status. Finally, our study assessed the impact of reading a book on sleep duration, good sleep quality, very good sleep efficiency and weight status comparing and contrasting this with the use of various EECDs which adds to its novelty.

## Conclusions

In conclusion, we have demonstrated that there may indeed be unappreciated effects of access to EECDs in the bedroom on sleep (duration, quality and efficiency) and weight status, more so, when EECDs are reportedly used during the hour before sleep. Our findings highlight the importance of ensuring that parents not only remove all EECDs from their children’s bedroom but also discourage their use during the hour before sleep. Alternatively, children should be encouraged to read a book during the hour before sleep as this may improve sleep duration, sleep quality and sleep efficiency and decrease prevalence of overweight and obesity.

## Abbreviations

BMI: Body Mass Index
CA$: Canadian dollars
EECD(s): Electronic entertainment and communication device(s)
OR: Odds ratio
REAL Kids: Raising healthy Eating and Active Living Kids
TTIB: Total time in bed
TV(s): Television(s)
